# Current forecast of COVID-19 in Mexico: A Bayesian and machine learning approaches

**DOI:** 10.1371/journal.pone.0259958

**Published:** 2022-01-21

**Authors:** Kernel Prieto

**Affiliations:** Instituto de Matemáticas, Universidad Nacional Autónoma de México, Mexico City, México; Italian National Research Council (CNR), ITALY

## Abstract

The COVID-19 pandemic has been widely spread and affected millions of people and caused hundreds of deaths worldwide, especially in patients with comorbilities and COVID-19. This manuscript aims to present models to predict, firstly, the number of coronavirus cases and secondly, the hospital care demand and mortality based on COVID-19 patients who have been diagnosed with other diseases. For the first part, I present a projection of the spread of coronavirus in Mexico, which is based on a contact tracing model using Bayesian inference. I investigate the health profile of individuals diagnosed with coronavirus to predict their type of patient care (inpatient or outpatient) and survival. Specifically, I analyze the comorbidity associated with coronavirus using Machine Learning. I have implemented two classifiers: I use the first classifier to predict the type of care procedure that a person diagnosed with coronavirus presenting chronic diseases will obtain (i.e. outpatient or hospitalised), in this way I estimate the hospital care demand; I use the second classifier to predict the survival or mortality of the patient (i.e. survived or deceased). I present two techniques to deal with these kinds of unbalanced datasets related to outpatient/hospitalised and survived/deceased cases (which occur in general for these types of coronavirus datasets) to obtain a better performance for the classification.

## 1 Introduction

Several mathematical models for disease transmission, and to predict and control disease spread have been proposed because emerging and re-emerging infectious diseases represent a major threat to public health, and may cause large economic and social losses. Vaccination is the principal control measure for reducing the spread of many infectious diseases [1, 2]. However, recent epidemics such as H1N1, Ebola, and MERS-CoV have required strong government interventions for fast eradication [3]. Based on previous pandemics, scientists have warned that another pandemic could strike at any moment. Therefore, a considerable effort has been made to study the impact of control measures to eradicate the outbreak of an epidemic, and in particular an immediate response for a possible influenza pandemic crisis [4]. Mathematical models include compartmental epidemic models, which are deterministic systems of ordinary and partial differential equations or stochastic difference equations [5]. For diseases such as influenza, typhoid fever, anthrax, diphtheria, tetanus, cholera, hepatitis B, pertussis, pneumonia, and coronavirus, the process of transmission between individuals takes place because of an initial inoculation of a small amount of pathogen units. The pathogen then reproduces quickly within the host during a period of time, which is called the incubation time. During this period, the pathogen affluence is enough to activate transmission to other susceptible individuals [6]. Many mathematical models assume that the disease incubation period is negligible once an individual is infected; that is, the infected individual becomes infectious instantaneously. The compartmental model based on these assumptions is known as the Susceptible-Infectious-Removed (SIR or SIRS) model [7], depending on whether the acquired immunity is permanent or temporal. For viral infections such as rubella and measles, the infected individual acquires permanent immunity. However, many diseases have an incubation (latent) period of time before the hosts become infectious, such as influenza, typhoid fever, anthrax, diphtheria, tetanus, cholera, hepatitis B, pertussis, pneumonia and coronavirus [8]. Meanwhile, diseases with a long immune period include polio, chicken-pox, whooping cough, smallpox and dengue fever. To take this incubation period of the disease into account, another population compartment, named exposed class, *E*, is incorporated into this type of model (i.e. SIR or SIRS). A susceptible individual who has just been infected first goes through the exposed class during the incubation period of the disease and the exposed individual then becomes infectious. The resulting models are of Susceptible-Exposed-Infectious-Removed (SEIR or SEIRS) type. I note that there is more literature on SIR and SEIR models than SIRS and SEIRS models; that is, those which permanent immunity is not assumed. I refer the reader to [9–11] for references on SEIRS models and [6, 12–16] for references on SEIR models.

Numerous efforts to forecast and produce mathematical control models for disease transmission have been proposed since the re-emergence of the coronavirus named SARS-CoV-2 [17–23]. The first coronavirus outbreak was named SARS-CoV (where SARS stands for severe acute respiratory syndrome), which caused a pandemic with a variety of incidences in 29 countries around the world. A Bayesian compartment model (SEIR: Susceptible, Exposed, Infected and Removed) was presented to study the spread of the first coronavirus in 2002 [24]. The mean incubation period was 5.3 days (95% Credible Interval 4.2 − 6.8 days), which is close to the latter coronavirus mean incubation period, which is reported as 5.1 [25]. In addition, the reported mean recovery period, from symptom onset to recovery, was 21 days (%95 Credible Interval 16 − 26 days), which is higher when compared to the second coronavirus recovery period, which is reported to be around 14 days [26]. The use of social distance as a control strategy for SARS was explored in [27]. The basic and effective reproductive numbers of SARS-Cov were estimated in [28]. In addition, a spatiotemporal analysis of SARS-CoV was presented in [29]. Another type of coronavirus emerged in 2015 in the Republic of Korea, which was named Middle East Respiratory Syndrome Coronavirus (MERS-CoV). Seventeen years after the first appearance of SARS-CoV (November, 2002), another virus strain has emerged; which is called SARS-CoV-2 or COVID-19. Many attempts to predict the dynamics of the coronavirus pandemic have been presented since the start of the second coronavirus outbreak in Wuhan City in December of 2019, some with a Bayesian inference approach [22, 30, 31]. A wide range of predictions have been presented in model calibrations using confirmed-case data because of the nonidentifiability in these models [32].

The rest of this paper is organised as follows. Section 2 describes the mathematical formulation of the contact tracing model for coronavirus disease and it outlines the Bayesian inference framework to predict the dynamics of its spread. Besides predicting the coronavirus cases, mathematical methods are used to forecast hospital care demand and mortality among patients with COVID-19 who present comorbidities related with COVID-19. I aimed to develop models of COVID-19 using Machine Learning to accurately predict both hospital care demand and mortality based on patients who present diseases such as hypertension, obesity, diabetes and smoking. These models and methods are presented in Section 4 using the dataset [33]. Each section presents the mathematical framework and numerical results. A discussion and some conclusions are presented in the last section 5.

## 2 Bayesian forecasting

### 2.1 Model formulation

Control strategies for infectious diseases include effective vaccination [34], early detection, proper treatment, isolation, quarantine, and educational campaigns. With the aim of studying the effect of contact tracing in the propagation of an infectious disease, I formulate a contact tracing model. Here, it is assumed that the disease transmits horizontally (i.e., vertical transmission is neglected). The horizontal transmission can occur either by direct contact (e.g., touching, licking, or biting) or by indirect contact with no physical contact (e.g., vectors or fomites).

The SIR and SEIR frameworks have been used in most current studies of COVID-19 transmission dynamics. Inspired by a full data-driven approach, I have tried to use all of the available reliable data to forecast the spread of the HIV-AIDS disease, keeping in mind that a simple model may fit better than complex models [32]. Next, I formulate a mathematical model considering isolation due to contact tracing as suggested in [6] and the models proposed in [27, 32, 35]. This model analyzes the significance of isolating the probable infected individuals. The total population, *N*, is divided into the following seven epidemiological classes SsEIQR: susceptible *S*, suspects (susceptible quarantined) *s*: people who have had contact with an infectious person or with someone who had contact with an infectious person), exposed *E*, people who have contracted the virus but are not yet infectious, the undetected infectives *A*, asymptomatic people, sick people reported in quarantine *I* (i.e., individuals are isolated at home or in the hospital), recovered people *R*, and the last state variable *P* denotes the deceased by coronavirus. I assume that the disease transmission rate, *λ*, is decomposed of two parts: the disease transmission rate by symptomatic people and by asymptomatic people; *λ* = *β*_*a*_ + *β*_*s*_. I assume that a fraction *q* of the contacts whom infected individuals have had recently are sought and isolated. I model contact tracing by forcing a fraction *q* of those who have recently had contact with an infectious individual to be quarantined, where they will spend an average 1/*τ* days. Importantly, I assume that these individuals are quarantined before they have a chance to generate any subsequent infection. Because of this latter assumption, contact tracing does not need to be recursive. The parameter *α*^−1^ and *γ*^−1^ represents the mean latent period and the recovery period, respectively. The parameter *ρ* represents the proportion between the symptomatic class and the asymptomatic class. Finally, the parameter *σ* denotes the death rate by the disease. The description of all the parameters of the contact tracing model proposes here is on Table 1. My suggested model reads as follows

$$\begin{array}{c} \begin{array}{cc} \frac{dS}{dt} & =-\frac{(\left(1-q\right)\beta_{s}I+\beta_{a}A)S}{N}-\frac{q\beta_{s}IS}{N}+\tau s\\ \frac{ds}{dt} & =\frac{q\beta_{s}IS}{N}-\tau s\\ \frac{dE}{dt} & =\frac{(\left(1-q\right)\beta_{s}I+\beta_{a}A)S}{N}-\alpha E\\ \frac{dA}{dt} & =\rho \alpha E-\gamma A\\ \frac{dI}{dt} & =(1-\rho )\alpha E-(\gamma +\sigma )I\\ \frac{dR}{dt} & =\gamma (A+I)\\ \frac{dD}{dt} & =\sigma I \end{array} \end{array}$$ (1)

**Table 1:** Parameters of the contact tracing model (1).

Parameter	Description	Value
*β*_*s*_	transmission rate of the disease by symptomatic individuals	to be estimated
*β*_*a*_	transmission rate of the disease by asymptomatic individuals	to be estimated
*ρ*	the fraction of asymptomatics/symptomatics	to be estimated
1/*γ*	recovery period (days)	to be estimated
*σ*	death rate by the disease	to be estimated
*q*	proportion of quarantined individuals by contact tracing	to be estimated
1/*τ*	period of quarantined (days)	14 [26]
1/*α*	latent period (days)	5.1 [25]
*E*_0_	initial condition for exposed class *E*(0)	to be estimated
*A*_0_	initial condition for asymptomatic class *A*(0)	to be estimated
*I*_0_	initial condition for symptomatic class *I*(0)	to be estimated

The total population *N*(*t*) is determined by *N*(*t*) = *S*(*t*) + *s*(*t*)+ *E*(*t*) + *A*(*t*) + *I*(*t*) + *R*(*t*) + *D*(*t*). I note that a more complex model is suggested in [6], considering stages in the exposed and infectious compartments but considering to decompose the force of transmission *λ*. In model (1), I have assumed that the compartment of suspect people are unexposed people of the disease during the quarantine period similarly to the quarantined compartment, *S*_*q*_, and the compartment, *E*_*q*_, of model proposed in [36, 37], respectively. A less similar compartment, *Q*, to my proposed compartment *s* is proposed in [38]. A more realistic version would be to consider that people during the quarantine period are exposed to be infected as in [39, 40]. Actually, in [39] is considered a parameter which measures the efficacy of quarantine to prevent the acquisition of infection by quarantined-susceptible individuals during the quarantine period. Finally, these articles Reviews [41, 42] analyze and categorize studies of quarantine through contact tracing.

Future work may explore the contact tracing model in [43], which proposes a very interesting and robust force of transmission *λ* that is dependent of time and with a delay. A sensitivity analysis shows that *λ* is the highest sensible parameter in this kind of compartment model. Therefore, it is very important to select this parameter adequately. Further interesting options for contact tracing models can be found in [44]. A robust review of contact tracing models can be found in [45] and quarantine models can be found in [46]. A detailed mathematical analysis of this type of SEIR model can be found in [47, 48].

## 3 Data

All code and data used to complete these simulations and analyses presented in section 2 based on the Stan Package, the *t- walk* Package is publically available on https://github.com/kernelprieto/COVID_MEX2, and https://github.com/kernelprieto/COVID_MEX1, respectively. All code and data used to complete the simulations and analyses presented in section 4 based on Machine Learning methods is publically available on https://github.com/kernelprieto/COVID-19_Comorbidities.

### 3.1 Parameter estimation

I used the daily updated data for the parameter estimation [33]. From the mathematical point of view, the parameter estimation of a system of ordinary differential equations is regarded as an inverse problem. The fitting curve or estimation of the parameters of a model is considered to be an inverse problem from the mathematical point of view. Typically, an optimisation method such as the Landweber in [49–53], or faster methods such as the Levenberg-Marquardt or Conjugate Gradient methods, and regularisation techniques, such as Tikhonov, Sparsity or Total Variation are used to solve this inverse problem. In this manuscript, I used Bayesian inference to solve the inverse problem because it is a tool that combines uncertainty propagation of measured data with available prior information of the model’s parameters. It is also a numerically more stable approach than classical methods that rely on the starting parameter point being relatively close to the true one, otherwise the solution obtained corresponds to a local minimum. Moreover, the classical methods only give a point estimate solution instead of a band of solutions using Bayesian inference; that is, in a Bayesian framework, one works with credible intervals. Some studies that have used Bayesian inference include [5, 18, 30, 34, 35, 54–60]. A Bayesian framework to model the spread of the first coronavirus (i.e., SARS-CoV) was presented in [24]. Using Bayesian inference, solutions of the inverse are obtained from the posterior distribution of the parameters of interest, and a solution of interest is obtained using the Maximum a Posterior (MAP). This MAP gives the parameter value for which the posterior density is maximal. I can also calculate the median and quantiles from this posterior sample. As previously mentioned, the Bayesian framework provides a natural and formal way to quantify the uncertainty of the quantities of interest. By denoting the state variable *x* = (*S*(*t*), *s*(*t*), *E*(*t*), *I*(*t*), *Q*(*t*), *R*(*t*), *P*(*t*)) ∈ (*L*^2^([0, *T*])^*n*^ (i.e., *n* denotes the number of state variables, here *n* = 7) and the parameters $\theta =\left(\beta ,q,\delta ,\alpha ,\gamma ,\sigma ,s\left(0\right),E\left(0\right),I\left(0\right),Q\left(0\right)\right)\in R^{m}$ (i.e., *m* denotes the dimension number of parameters to estimate, here *m* = 10), I can write the model (1) as the following initial value problem

$$\begin{array}{ccc} \dot{x} & =\varphi (x,\theta ) & \left(2\text{a}\right)\\ x(0) & =x_{0}. & \left(2\text{b}\right) \end{array}$$

Problem (2), defines a mapping Φ(*θ*) = *x* from parameters *θ* to state variables *x*, where $\Phi :R_{+}^{m}\to {\left(L^{2}(\left[0,T\right]\right)}^{n}$, where $R_{+}$ denotes the nonnegative real numbers. I assume that Φ has a Fréchet derivative; that is, the mapping $F^{\prime }\left(\theta \right):R_{+}^{m}\to {\left(L^{2}\left(\left[0,T\right]\right)\right)}^{n}$, is injective. Thus, the forward problem (2) has a unique solution *x* for a given *θ*. The Fréchet derivative of Φ, denoted by Φ′, results to be the usual derivative for the system (1) because the domain and range of Φ′ are finite dimensional spaces. Usually, not all states of the system can actually be directed measured (i.e., the data consists of measurements of some state variables at a discrete set of points *t*_1_, …, *t*_*k*_); for example, in epidemiology, these data consist of number of cases of confirmed infected people. This defines a linear observation mapping from state variables to data $\Psi :{\left(L^{2}\left(\left[0,T\right]\right)\right)}^{n}:\to R^{s\times k}$, where *s* ≤ *n* is the number of observed variables and *k* is the number of sample points. Let $F:R^{m}\to R^{s\times k}$ be defined by *F*(*θ*) = Ψ(Φ(*θ*)), which is called the forward problem. The inverse problem is formulated as a standard optimisation problem

$$\begin{array}{c} \underset{\theta \in R^{m}}{\text{min}}{∥F\left(\theta \right)-y_{\text{obs}}∥}^{2}, \end{array}$$ (3)

such that *x* = Φ(*θ*) holds, with *y*_obs_ is the data that has error measurements of size *η*. Problem (2) may be solved using numerical tools to deal with a nonlinear least-squares problem or the Landweber method, or a combination of both. I implement Bayesian inference to solve the inverse problem (3) in this manuscript. From the Bayesian perspective, all of the state variables *x* and parameters *θ* are considered as random variables and the data *y*_obs_ is fixed. For random variables *x*, *θ*, the joint probability distribution density of data *x* and parameters *θ*, denoted by *π*(*θ*, *x*), is given by *π*(*θ*, *x*) = *π*(*x*|*θ*)*π*(*θ*), where *π*(*x*|*θ*) is the conditional probability distribution, which is also called the likelihood function, and *π*(*θ*) is the prior distribution, which involves the prior information of parameters *θ*. Given *x* = *y*_obs_, the conditional probability distribution *π*(*θ*|*y*_obs_), which is called the posterior distribution of *θ*, is given by the Bayes’ theorem:

$$\begin{array}{c} \pi \left(\theta |y_{\text{obs}}\right)\propto \pi \left(y_{\text{obs}}|\theta \right)\pi \left(\theta \right), \end{array}$$ (4)

If additive noise is assumed:

$$\begin{array}{c} y_{\text{obs}}=F\left(\theta \right)+\eta , \end{array}$$

where *η* is the noise due to discretisation, model error and measurement error. If the noise probability distribution *π*_*H*_(*η*) is known, and *θ* and *η* are independent, then

$$\begin{array}{c} \pi \left(y_{\text{obs}}|\theta \right)=\pi_{H}\left(y_{\text{obs}}-F\left(\theta \right)\right). \end{array}$$

All of the available information regarding the unknown parameter *θ* is codified into the a prior distribution *π*(*θ*), which specifies our belief in a parameter before observing the data. All of the available information regarding how I obtained the measured data is codified into the likelihood distribution *π*(*y*_obs_|*θ*). This likelihood can be seen as an objective or cost function because it punishes deviations of the model from the data. To solve the associated inverse problem (4), one may use the maximum a posterior (MAP)

$$\begin{array}{cccc} \theta_{\text{MAP}} & =\underset{\theta }{\text{max}}\pi \left(\theta |x\right), & \theta_{\text{CM}} & =E[\pi (\theta |x)]. \end{array}$$

I used the dataset $y_{\text{obs}}=\left(\tilde{s},\tilde{Q},\tilde{P}\right)$, which correspond to the suspects, diagnosed sick cases and the deceased, respectively. I note that I have not used the data column corresponding to the recovered here because this data was not been collected in a large range (from the beginning) of days. A Poisson distribution with respect to the time is typically used to account for the discrete nature of these counts. However, the variance of each component of the dataset *y*_obs_ is larger than its mean, which indicates that there is over-dispersion of the data. Thus, it is more appropriate to use the Negative Binomial likelihood distribution because it has an additional parameter that allows the variance to exceed the mean [34, 60, 61]. In fact, the Negative Binomial is a mixture of Poisson and Gamma distributions, where the rate parameter of the Poisson distribution itself follows a Gamma distribution [61, 62]. Here, I have used the following expression for the Negative Binomial distribution

$$\begin{array}{c} NB\left(y|\mu ,\phi \right)=\frac{\Gamma (y+\phi )}{\Gamma (y)\Gamma (\phi )}{\left(\frac{\mu }{\mu +\phi }\right)}^{y}{\left(\frac{\phi }{\mu +\phi }\right)}^{\phi }, \end{array}$$ (5)

where *μ* is the mean of the random variable y∼NB(y|μ,ϕ) and *ϕ* is the overdispersion parameter; that is,

$$\begin{array}{cccc} E[Y] & =\mu , & \text{Var}(Y) & =\mu +\frac{\mu^{2}}{\phi }. \end{array}$$

I recall that Poisson distribution has mean and variance equal to *μ*, so *μ*^2^/*ϕ* > 0 is the additional variance of the negative binomial with respect to the Poisson distribution. Therefore, the inverse of the parameter *ϕ* controls the overdispersion. This is important when selecting its support for parameter estimation. In addition, there are alternative forms of the Negative Binomial distribution. In fact, I have used the first option *neg_bin* of the Negative Binomial distribution of Stan [63]. I acknowledge that some scientists have had success with the second alternative representation of the NB distribution [58]. I assume independent Negative Binomial distributed noise *η*; that is, all dependency in the data is codified into the contact tracing model. In other words, the positive definite noise covariance matrix *η* is assumed to be diagonal. Therefore, using Bayes formula, the likelihood is

$$\begin{array}{c} \pi \left(\theta |\tilde{s},\tilde{I},\tilde{D}\right)\propto \pi \left(\tilde{s}|\theta \right)\pi \left(\tilde{I}|\theta \right)\pi \left(\tilde{D}|\theta \right)\pi \left(\theta \right). \end{array}$$

As mentioned earlier, I approximate the likelihood probability distribution corresponding to suspects, diagnosed cases, and deaths with a Negative Binomial distribution

$$\begin{array}{cccccc} {\tilde{s}}_{i} & ∼NB(s_{i}\left(\theta \right),\phi_{0}^{2}), & {\tilde{I}}_{i} & ∼NB(I_{i}\left(\theta \right),\phi_{1}^{2}), & {\tilde{D}}_{i} & ∼NB(D_{i}\left(\theta \right),\phi_{2}^{2}), \end{array}$$

where the index *i* denotes the number time, in our case the number of days, and *ϕ*_0_, *ϕ*_1_ and *ϕ*_2_ are the parameters corresponding to the overdispersion parameter of the Negative Binomial distribution (5), respectively, of each data component.

For independent observations, the likelihood distribution *π*(*y*|*θ*), is given by the product of the individual probability densities of the observations

$$\begin{array}{c} \pi \left(y_{\text{obs}}|\theta \right)=\prod_{i=1}^{n}\pi \left({\tilde{s}}_{i}|\theta \right)\pi \left({\tilde{I}}_{i}|\theta \right)\pi \left({\tilde{D}}_{i}|\theta \right), \end{array}$$

where the mean *μ* of the negative binomial distribution $NB(I_{i}\left(\theta \right),\phi_{1}^{2})$, is given by the solution *I*(*t*) of the model (1) at time *t* = *t*_*i*_. Analogously, the mean for the negative binomial distributions $NB(s_{i}\left(\theta \right),\phi_{0}^{2})$ and $NB(D_{i}\left(\theta \right),\phi_{2}^{2})$ are the solutions *s*(*t*) and *D*(*t*) of (1) at time *t*_*i*_, respectively. For the prior distribution, I select LogNormal distribution for the *β* parameter and Uniform distributions for the rest of parameters to estimate: *q*, *δ*, *α*, *γ*, *σ*, *s*_0_, *E*_0_, *I*_0_, *Q*_0_.

$$\begin{array}{cc} \pi (\theta ) & =\prod_{i=1}^{n}LN\left(a_{\beta },b_{\beta }\right)U\left(a_{q},b_{q}\right)U\left(a_{\delta },b_{\delta }\right)U\left(a_{\alpha },b_{\alpha }\right)U\left(a_{\gamma },b_{\gamma }\right) \end{array}$$ (6)

$$\begin{array}{c} \;\times U\left(a_{s_{0}},b_{s_{0}}\right)U\left(a_{E_{0}},b_{E_{0}}\right)U\left(a_{I_{0}},b_{I_{0}}\right)U\left(a_{Q_{0}},b_{Q_{0}}\right). \end{array}$$ (7)

**Fig 1:**
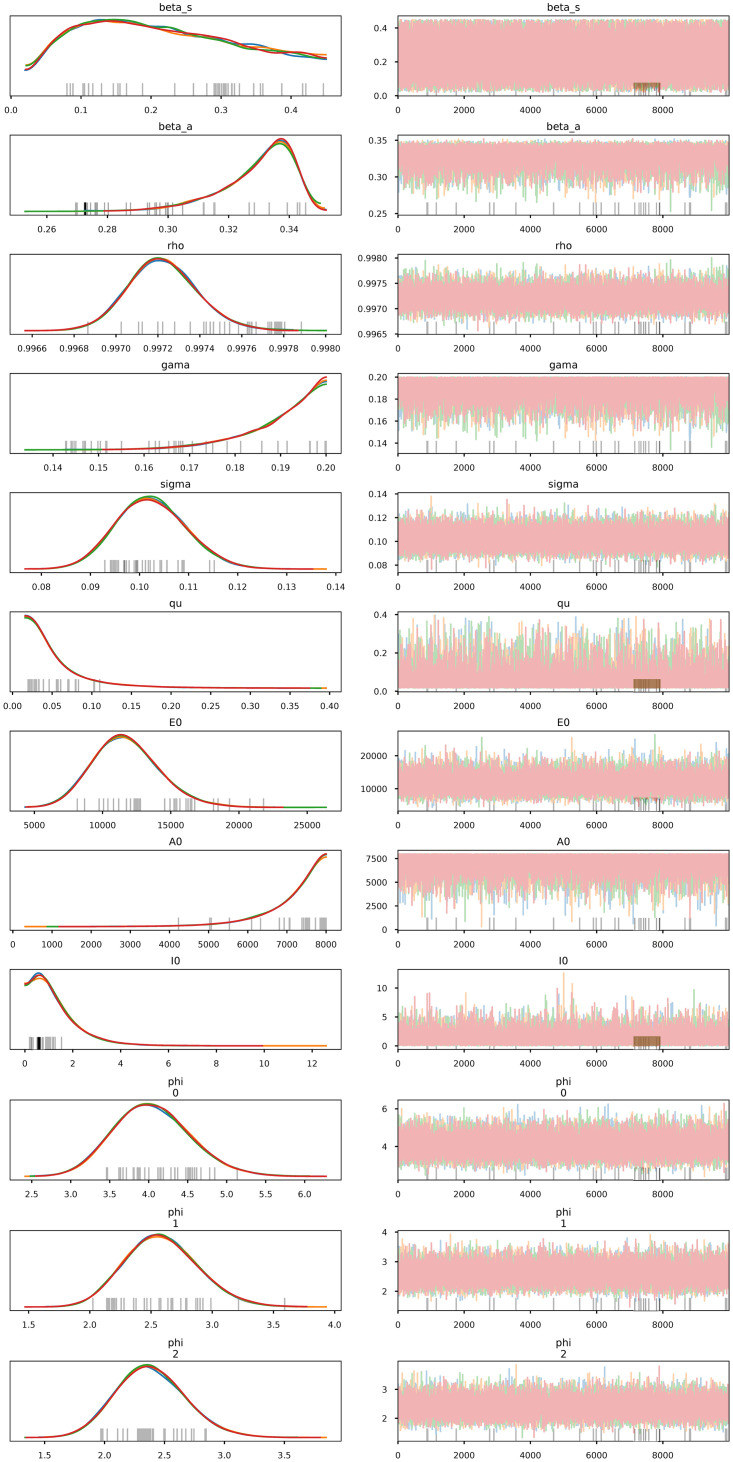
Credible intervals of parameters of model (1). Credible intervals within 95% Highest-Posterior Density (HPD).

**Fig 2:**
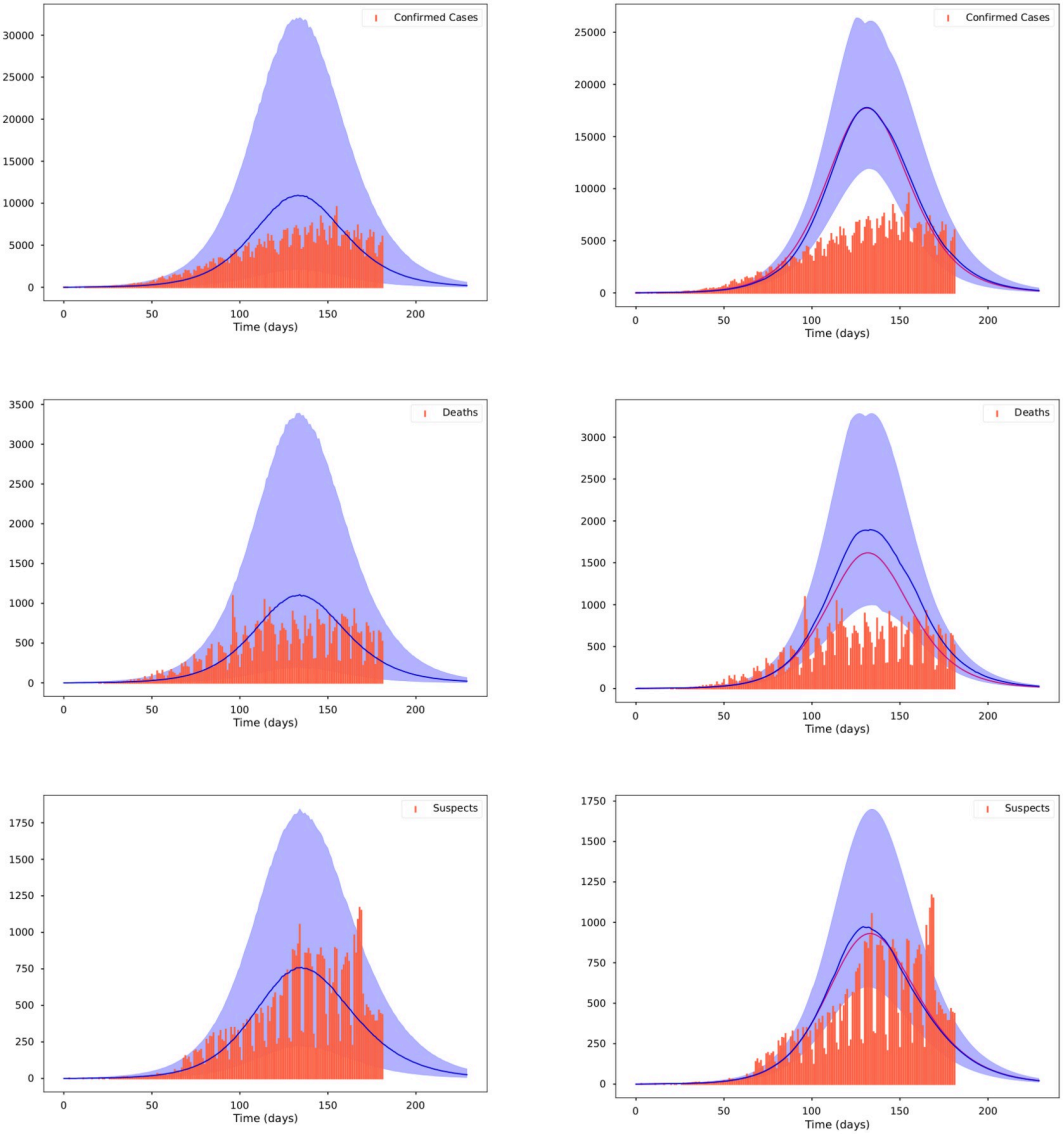
Incidence analysis for Mexico considering data for the first 182 days of the pandemic (until 6 August 2020). Left column corresponds to the inference analysis using the Stan Package. Right column corresponds to the inference analysis using the *t- walk* Package. Row from top to bottom correspond to the confirmed cases, deceases and suspects. Posterior uncertainty is illustrated with the blue shadow areas within the 95% Highest-Posterior Density. Red bars correspond to the data, i.e., the confirmed cases, deceases and suspects. Blue line denotes the median, and the purple line on the right column correspond to the mode.

**Fig 3:**
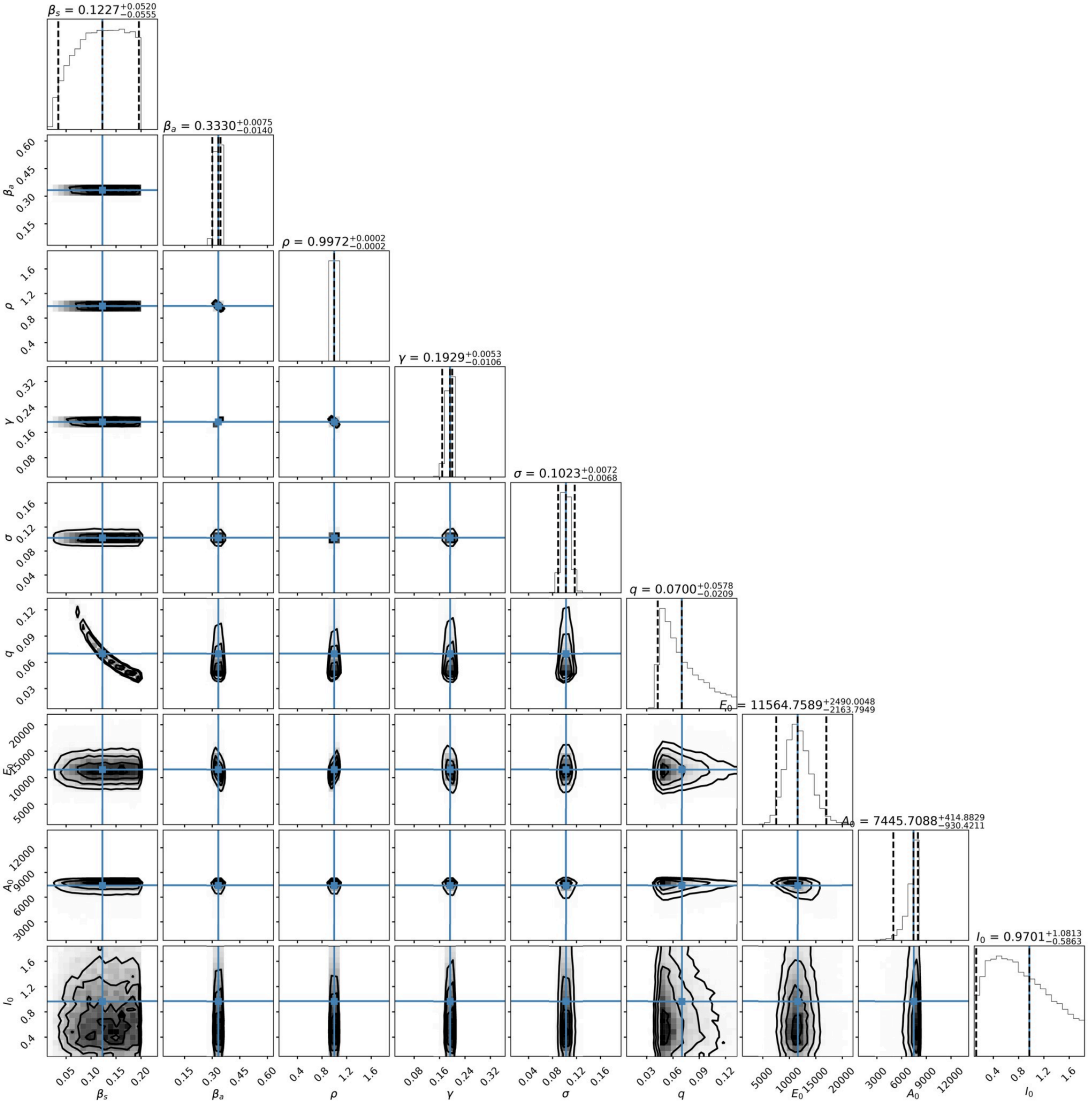
Joint probability density distributions of the estimated parameters. Joint probability density distributions of parameters of model (1) within 95% HPD using the Stan Package [63]. The blue lines represent the medians.

**Fig 4:**
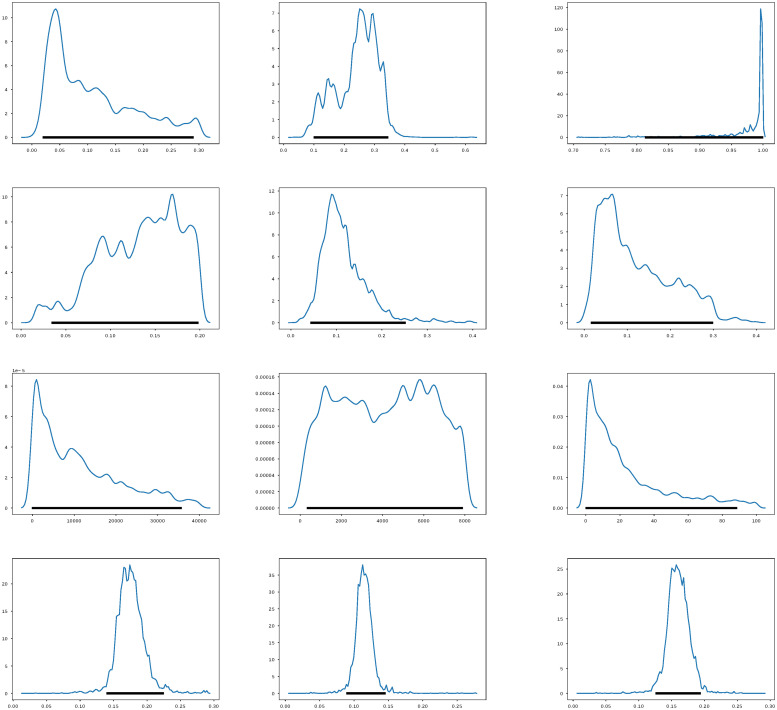
Credible intervals for the estimated parameters. Credible intervals for the estimated parameters of model (1) within %95 of HPD using the *t- walk* Package [64]. Top row from left to right, the parameters: *β*_*s*_, *β*_*a*_, *ρ*. Middle row from left to right: *γ*, *σ*, *q*. Middle row from left to right: *E*_0_, *A*_0_, *I*_0_. Bottom row from left to right: *ϕ*_0_, *ϕ*_1_, *ϕ*_2_.

The posterior distribution *π*(*θ*|*y*_obs_) given by (4) does not have an analytical closed form because the likelihood function, which depends on the solution of the nonlinear SsEAIRD model, does not have an explicit solution. Then, I explore the posterior distribution using the Stan Statistics package [63], general purpose Markov Chain Monte Carlo Metropolis-Hasting (MCMC-MH) algorithm to sample it, the package *t- walk* [64]. Both algorithms generate samples from the posterior distribution *π*(*θ*|*y*_obs_) that can be used to estimate marginal posterior densities, mean, credible intervals, percentiles, variances, and so on. I refer the reader to [65] for a more complex description of MCMC MH algorithms. The dataset in [33] contains the information regarding the number of diagnosed cases, deaths, and suspects. Figs 1–3 show the results of forecasting the disease using the Stan package [63]. Fig 1 show the credible intervals of parameters of model (1) within 95% Highest-Posterior Density. Fig 1 shows the results of six chains of the MCMC MH algorithm. Fig 2 shows the incidence analysis for Mexico considering data for the first 182 days of the pandemic. Left column corresponds to the inference analysis using the Stan Package. Right column corresponds to the inference analysis using the *t- walk* Package. Row from top to bottom correspond to the confirmed cases, deceases and suspects. Posterior uncertainty is illustrated with the blue shadow areas within the 95% Highest-Posterior Density. Red bars correspond to the data, i.e., the confirmed cases, deceases and suspects. Blue line denotes the median, and the purple line on the right column correspond to the mode. Fig 3 shows Joint probability density distributions of parameters of model (1) within 95% HPD using the Stan Package [63]. The blue lines represent the medians. Table 2 shows the parameter estimated using the Stan package with the quantiles 2.5%, 25%, 50%, 75%, 97.5%. I perform 20000 iterations, with 10000 of them as a burn-in. I have used the interface in Python (PyStan). I have used the Hamilton Monte Carlo and No-U-Turn Sampler (NUTS) algorithms, obtaining similar performance. I point out that using Automatic Differentiation Variational Inference (ADVI) is much faster than the previously mentioned algorithms, with very similar results. Fig 4 and the right-hand column of Fig 2 show corresponding results using the *t- walk* package (the Python version). Fig 4 shows the credible intervals for the estimated parameters of model (1) within %95 of HPD using the *t- walk* Package [64]. Top row from left to right, the parameters: *β*_*s*_, *β*_*a*_, *ρ*. Middle row from left to right: *γ*, *σ*, *q*. Middle row from left to right: *E*_0_, *A*_0_, *I*_0_. Bottom row from left to right: *ϕ*_0_, *ϕ*_1_, *ϕ*_2_ I performed 600000 iterations with 300000 of them as burn-in. Using both packages, I have made predictions until the day 240, meaning 16 October. Future work will analyze the identifiability of the parameters of model (1), as suggested in [59, 66, 67], specifically the *ρ* parameter, because this parameter is multiplied by the period of incubation of the disease, *α*. Thus, estimating both parameters simultaneously may lead to the nonidentifiability difficulty. In this work, I have assumed the value for the period of incubation of the disease given, equal to 5.1 days [25].

**Table 2:** Estimation of the parameters of the model (1).

	mean	2.5%	25%	50%	75%	97.5%
*β*_*s*_	0.222620	0.044925	0.128393	0.211603	0.312559	0.433075
*β*_*a*_	0.329556	0.299295	0.323674	0.332539	0.338223	0.344950
*ρ*	0.997225	0.996923	0.997114	0.997219	0.997329	0.997565
*γ*	0.190190	0.167331	0.186027	0.192736	0.196920	0.199693
*σ*	0.102499	0.089572	0.097630	0.102195	0.107137	0.116798
*q*	0.056694	0.019540	0.027548	0.040514	0.066996	0.193501
*E*_0_	11780.966835	7526.224012	10093.482221	11627.748681	13273.949132	17010.362332
*A*_0_	7182.017997	4917.603144	6872.829362	7445.705247	7765.232788	7977.776073
*I*_0_	1.220856	0.130659	0.540569	0.970296	1.627982	3.748581
*ϕ*_0_	4.048778	3.175233	3.712320	4.025514	4.363766	5.062059
*ϕ*_1_	2.589235	2.060252	2.384828	2.576913	2.778927	3.192292
*ϕ*_2_	2.389033	1.868520	2.191568	2.373326	2.573794	2.989858

Posterior estimation for the parameters of the contact tracing model (1) using the Stan Package [63]. First column correspond to the mean, then the respective percent of the Highest-Posterior Density.

## 4 Clinical analysis with machine learning

**Fig 5:**
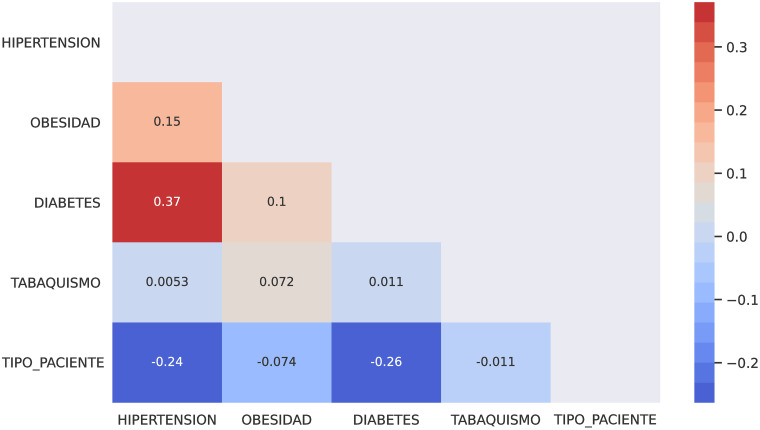
Covariance matrix of the most relevant chronic diseases with respect to the type of patient (outpatient/hospitalized) in Mexico.

**Fig 6:**
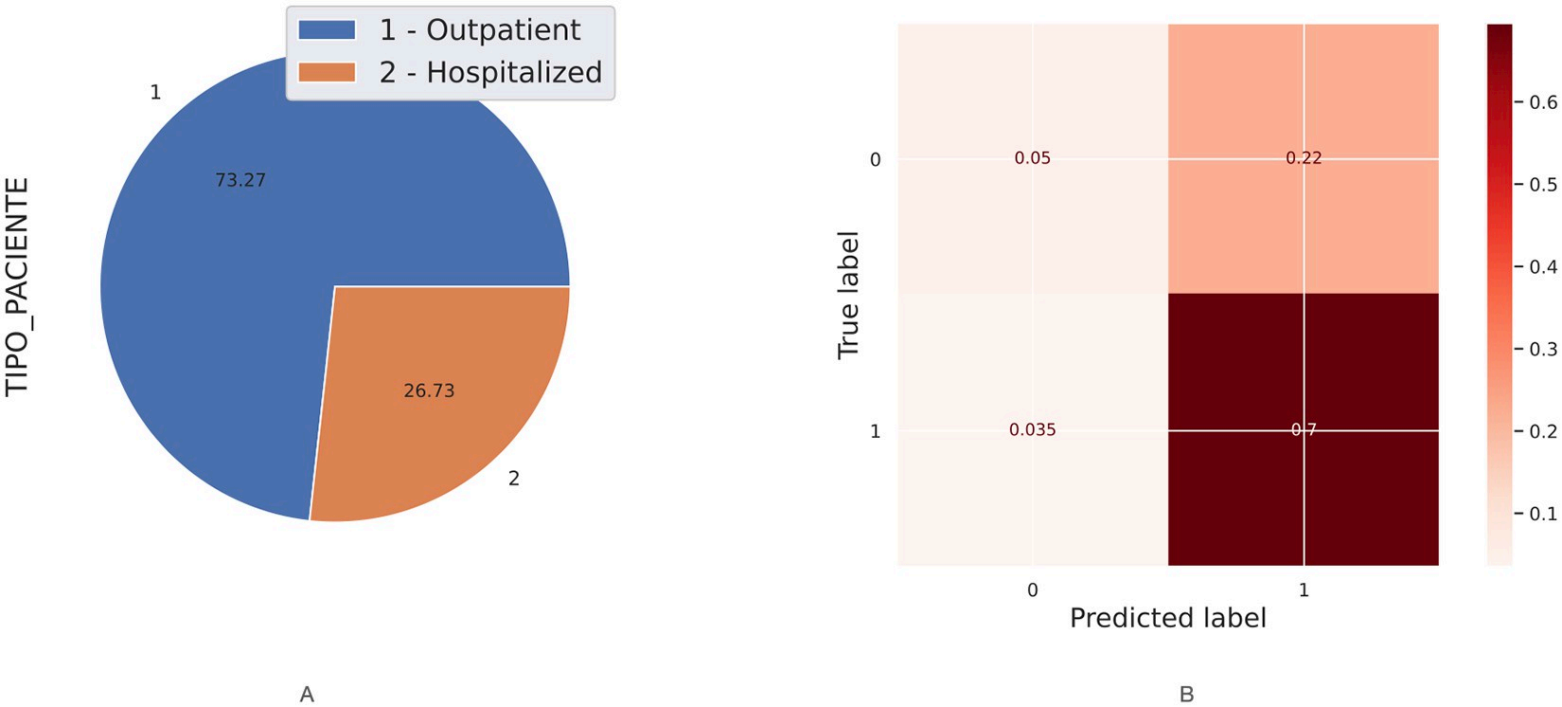
Analysis of outpatient versus hospitalized patients. (A): Percent relation between outpatient and hospitalized patients. (B): Confusion matrix using classical Machine Learning Methods.

**Fig 7:**
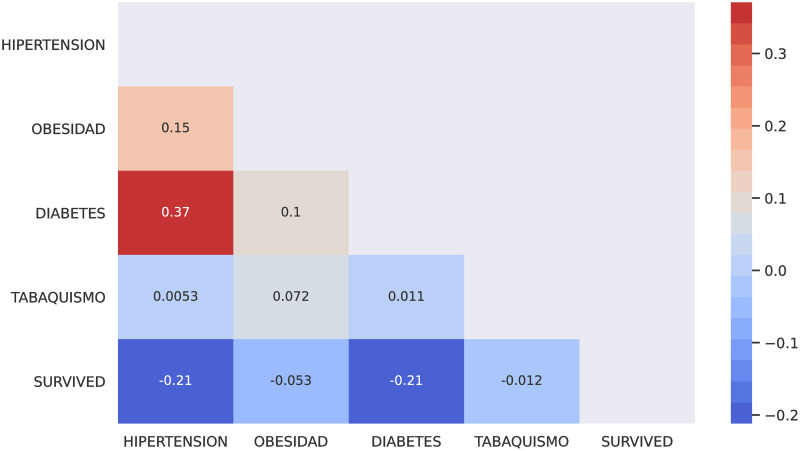
Covariance matrix of the most relevant chronic diseases with respect to the survival chance of a hospitalized patient in Mexico.

**Fig 8:**
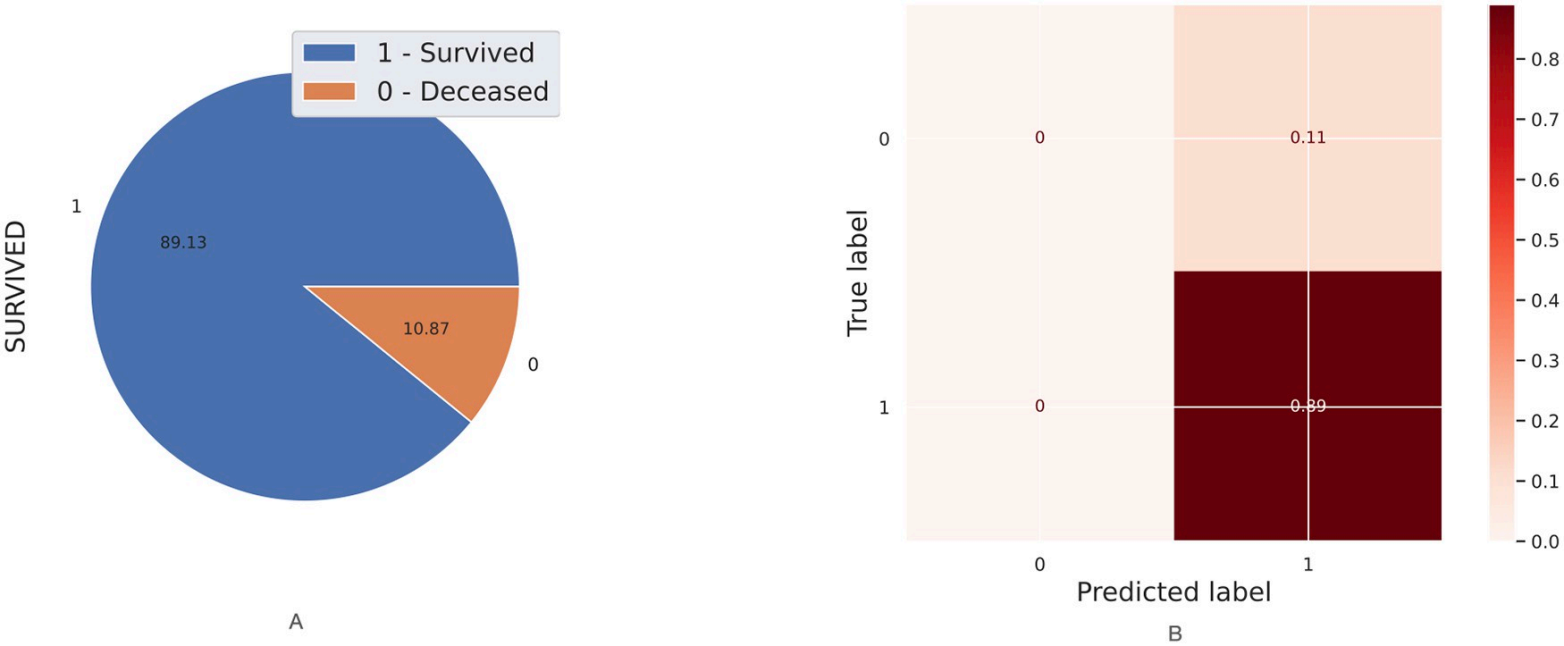
Analysis of survived versus deceased patients. (A): Percent relation between survived and deceased patients. (B): Confusion matrix using classical Machine Learning Methods.

In this section, I describe the methods to predict both hospital care and mortality using Machine Learning based on patients who have been diagnosed with morbidities such as hypertension, obesity, diabetes and smoking. Thus, I describe the comorbidity associated with coronavirus in Mexico using the [33] dataset. I have performed Machine Learning techniques on it as follows. First, I implemented a predicted classifier for the kind of patient, a person already diagnosed with coronavirus and who has got one or more of the most relevant chronic diseases (i.e., hypertension or diabetes). I have used prediction methods in Machine Learning, such as Logistic Regression, Decision Tree, and K-Neighbors classifiers, the naive Bayes (Bernoulli), and even the powerful methods such as XGBoost and Random Forest through the SciKit-learn package. Fig 5 shows the covariance matrix of the most relevant chronic diseases with respect to the two types of patient: outpatient or hospitalised patient. I can observe in Fig 5 that the most relevant chronic diseases with respect to the type of patient(outpatient or hospitalised) who has been diagnosed with coronavirus in Mexico are hypertension and diabetes. Table 3 shows the contingency table of these two chronic diseases with respect to the type of patient. Table 4 shows the contingency table of these two chronic diseases with respect to the patient’s survival possibility. Fig 6A shows the relationship in percent between outpatients and hospitalised patients. Fig 6B shows the confusion matrix result using classical Machine Learning Methods. I could add more characteristics such as age(range) to obtain more true negative cases because the differences in proportion of outpatient and hospitalised decreases. Next, instead of considering the type of patient (outpatient and hospitalised), I consider if the patient survives or dies once diagnosed with coronavirus. Fig 7 shows the covariance matrix of the most relevant chronic diseases with respect to the two types of patient: survived or deceased. One can see Fig 7 that the most relevant chronic diseases with respect to the survival of a person who has been diagnosed with coronavirus in Mexico are hypertension and diabetes. Fig 8A shows the relationship in percent between outpatients and hospitalised patients. Fig 8B shows the confusion matrix result using Logistic Regression. We point out that similar results are obtained using other Machine Learning methods such as Decision Tree, and K-Neighbors, XGBoost and Random Forest. By adding more characteristics such as age (range), one obtains similar results to Fig 8B; that is, one obtains zero true negative predictions. I remind the reader that false negatives and false positives are the two type of errors of rejecting the hypothesis when it was actually true and accepting the hypothesis when it was actually false. Under different circumstances, one type of error may be more critical than the other. For example, diagnosis of cancer would rather accept false positives than false negatives. The main difficulty in trying to predict if a person will survive assuming that they have either hypertension or diabetes is the rather unbalanced proportion between the two classes: survived and deceased. Unbalanced data is assumed with a category less than 20 percent. The lethality of coronavirus in the world is typically not greater than 15 percent.

**Table 3:** Contingency table of patient outpatient versus hospitalized.

Hipertension	Diabetes	Hospitalized	Outpatient
0	0	0.1871	0.8128
0	1	0.4757	0.5242
1	0	0.4069	0.5930
1	1	0.5846	0.4153

First two columns correspond the most relevant comorbidities with respect to COVID-19 in Mexico against the type of patient: outpatient and hospitalized using data [33].

**Table 4:** Contingency table of the survival of a hospitalized patient.

Hipertension	Diabetes	Deceased	Survived
0	0	0.0626	0.9374
0	1	0.2068	0.7931
1	0	0.1936	0.8063
1	1	0.3035	0.6964

First two columns correspond the most relevant comorbidities with respect to COVID-19 in Mexico against the chance of survival of a hospitalized patient using data [33].

As can be seen in Fig 8B, the true positives are very high but the prediction of true negatives is zero. I propose two options to deal with this difficulty. First, I have created a naive Bayes Multi-variate Bernoulli algorithm from scratch, as suggested in [68]. This algorithm was originally proposed as an anti-spam email filter. Analogous to their description of how to classify spam emails, a person with vector *x* = 〈*x*_1_, …, *x*_*m*_〉; that is, with multiple features but each one is assumed to be a binary-valued variable. In the case of comorbidity, *x* represents the type of disease. The decision rule for Bernoulli naive Bayes is based on the probability that a vector *x* belongs in category *c*:

$$\begin{array}{c} p\left(c|x\right)=\frac{p(c)p(x|c)}{p(x)}. \end{array}$$ (8)

**Fig 9:**
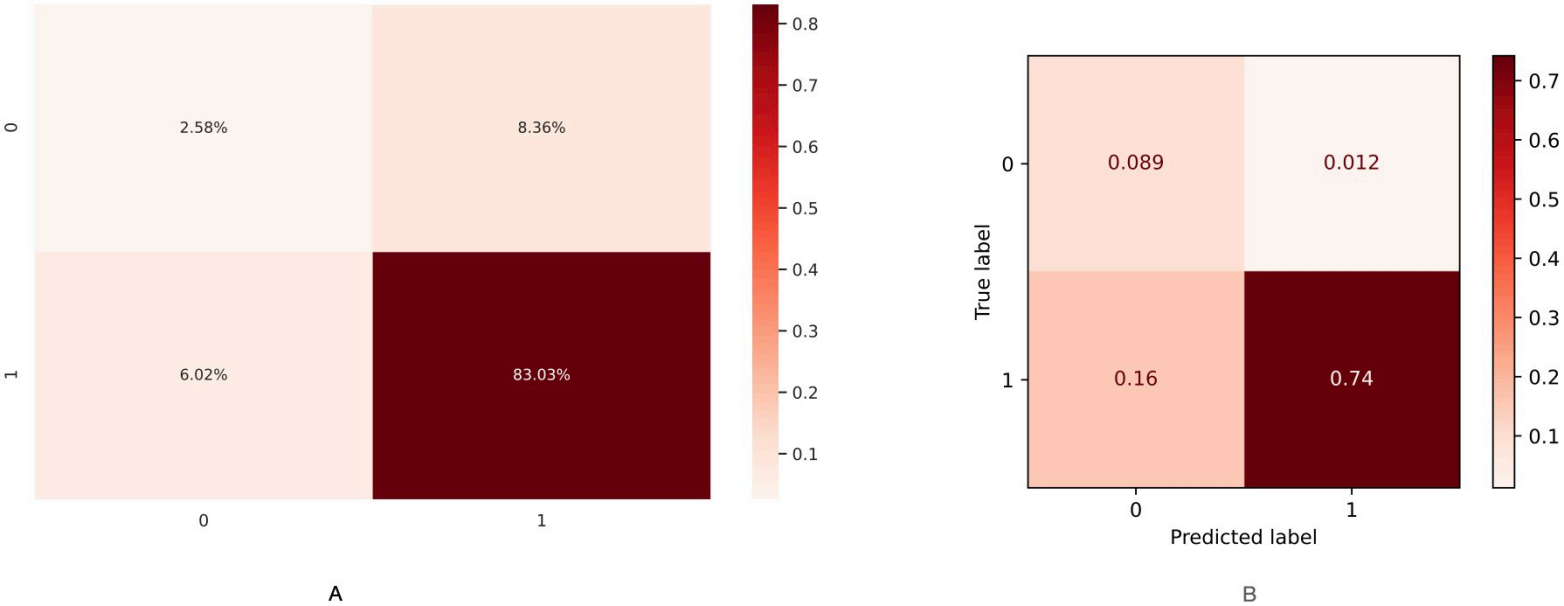
Improved prediction for the survival chance of a hospitalized patient. (A): Confusion matrix applying Naive Bayes with threshold *δ* = 0.45. (B): Confusion matrix using the SMOTE.

Given that the denominator does not depend on the category, NB classifies each “message” in the category that maximises the numerator in (8); that is, *p*(*c*)*p*(*x*|*c*). In the case of a “spam filter”, this is equivalent to classifying a message as spam whenever:

$$\begin{array}{c} \frac{p\left(c_{s}\right)p\left(x|c_{s}\right)}{p\left(c_{s}\right)p\left(x|c_{s}\right)+p\left(c_{h}\right)p\left(x|c_{h}\right)}>\delta , \end{array}$$ (9)

with *δ* = 0.5, where *c*_*h*_ and *c*_*s*_ denote the ham and spam categories. The important part doing this algorithm from scratch is that I can vary *δ* to obtain more true negatives at the expense of true positives, or vice versa. In our case, I increased the true negatives, the number of true positives are very high using whatever classifier is mentioned. Consequently, I can tune the threshold number of acceptance on the following formula 9. I selected *δ* = 0.45 (instead of 0.5) and obtained the following confusion matrix. Fig 9A shows the confusion matrix result using the Naive Bayes method, the percent of true negatives has increased approximately to 2.6, and the false negative has decreased, although the false negative has also increased.

Second, I propose to use the Synthetic Minority Oversampling Technique (SMOTE) function to balance the minority class (people who passed away due to coronavirus). SMOTE briefly consists of synthesising elements for the minority class, based on those that already exist. This works randomly by picking a point from the minority class and then computing the k-nearest neighbors for this point. The synthetic points are added between the chosen point and its neighbors. Fig 9B shows the result using the SMOTE technique. Another filter to predict the survival/mortality of an individual apart from the age of the patient, could be if the patient is already admitted to the hospital, this could result in having not a fully unbalanced dataset.

## 5 Discussion and conclusions

In section 2, I formulate a contact tracing model for the transmission of the COVID-19 and forcast the number of coronavirus cases using Bayesian inference based on two independent software packages: the Stan package [63] and the *t- walk* package [64]. Future work should address the identifiability of the parameters of model (1), as suggested in [59, 66, 67], specifically the *ρ* parameter, because this parameter is multiplied by the period of incubation of the disease, *α*. Thus, estimating both parameters simultaneously may lead to the nonidentifiability difficulty. In this work, I have assumed the value for the period of incubation of the disease given, equal to 5.1 days [25]. The value estimated for the parameter, *ρ*, which refers to the proportion of symptomatics and asymptomatics, was around.99, which indicates that a large percent are asymptomatic to this disease. This values is rather high compared with other results nowadays in the literature. This could due to the fact that it was assumed the value of incubation known, and this value could be incorrect for Mexico. I show trace plots, credible intervals, bands projections with medians and a MAP curve (for the *t- walk* case) and the joint crosstab probability distributions given as a corner. From Fig 2, I can observe that the government of Mexico took some measures to control the transmission of the disease. The model has many implicit assumptions which may be incorrect, e.g., it assumes that the transmission rate is constant and homogeneous through the whole country, which is by far incorrect [34], that is, we can certainly say that every region state in Mexico has its own pandemic, and it is not true that mobility from the North to the South in Mexico is the same as in a specific state of Mexico. A better projection for Mexico City, which has a considerable percentage of coronavirus in the whole country can be found in [69]. Also, the model does not take into account the government interventions, which in each state were announced by a color of the traffic light, red meaning almost all the activities had to be suspended, yellow, some of the activities could reactivate, and green, a considerable percentage of activities could reactivate, depending of each state government. These interventions could be added in the transmission rate in model (1) as in [35]. Despite this, the contact tracing model proposed here could be useful for public health to have a big picture how the pandemic is developing in the country. Also, if a efficient surveillance system is implemented in a pandemic, i.e., where suspects are traced and counted with a small uncertainty, this model could be rather useful for Health systems to make appropriate interventions. Another asset of the current model proposed is that it is simple and computationally efficient.

In section 4, I explore methods using Machine Learning to predict the hospital care demand and mortality based on patients who have been diagnosed with comorbidities with COVID-19. Firstly, the most relevant comorbidities with COVID-19 associated with both hospital care demand and mortality are hypertension and diabetes. Observing the confussion matrix of the predictor for the hospital care demand or the type of patient of coronavirus, mostly true positives (outpatient) 70% are predicted well, but a small percentage 5% of true negatives (hospitalized) are predicted well, moreover, a considerable 22% of false positives is obtained and a small 3.5% of false negatives. Thus, from around a 26% of hospitalized patients (Fig 6), I can predict well only a 5% of the patients who need hospital care. Also, on the one hand, the error type II, i.e., the false positives, is rather big, meaning that using this binary classifier, I would send home people who indeed needed hospital care. On the other hand, the false negatives is small, 3.5%, meaning that I incorrectly send patients to the Hospital when they indeed do not need Hospital care, taking rest at home and following Doctor’s advises would be enough. Under different circumstances, one type of error (type I or II) may be more critical than the other. If the hospital occupancy is relatively high, e.g., equal or higher than 80%, having a high number of false negatives would be risky since the Hospital could collapse. Otherwise, having a high number of false positive would be preferable instead of having false negatives. This projection inaccuracy is due to the unbalanced on the data related with the outpatients versus hospitalized ones. Although, there is no fully unbalanced, this dataset present a considerable majority of outpatients with respect hospitalized people.

Something worse happens when trying to predict the mortality patients with COVID-19, only true positives (survived ones) 89% can be predicted well, and a 0% of true negatives (deceaced ones) can be predicted, and significant error type II, false positives one obtains, i.e., one would give to 11% of people, a survivable expectancy when in fact, they will decease. This projection inaccuracy again is due to the unbalanced on the data related with the outpatients versus hospitalized ones since the lethality of coronavirus in the world is typically not greater than 15 percent. Therefore, I present two methods to deal with unbalanced data because it is the first case of a coronavirus dataset in the world, especially for the case of survived/deceased: first, I propose to use the Naive Bayes method; and second, I propose to use the SMOTE technique. Using the Naive Bayes method leads to a decrease of true positives to 83% (before was 89%) but obtaining a nonzero true negatives percentage 2.58%, also the false positives decreased to the value of 8.36% (before was 11%) and the false negatives increased to a nonzero value of 6.02% (before was 0%). As it was mentioned above, if the hospital occupancy is equal or higher than 80%, having a high number of false negatives would be risky since the Hospital could collapse. Otherwise, having a high number of false positive would be preferable instead of having false negatives. In case of using the SMOTE technique leads to a decrease of true positives to 74% (before was 89%) but obtaining a nonzero true negatives percentage 8.9%, which is rather significant since the proportion of people who survived and deceased is 89.13% versus 10.87%. Also the false positives decreased to the value of 1.2%(before was 11%) and the false negatives increased to a nonzero value of 16.0% (before was 0%). Thus, the value of false negatives obtained using the SMOTE technique is 2.65 times greater than the false negatives value obtained using the Naive Bayes method. As it was explained, unless the hospital occupancy is higher than 80%, it is less risky to have a bigger number of false positive than false negatives.

## Supporting information

S1 File (CSV)

S2 File (CSV)

S3 File (XLSX)

S4 File (XLSX)
